# Optimizing hydration in Chinese youth taekwondo athletes: evidence-based daily and training session-specific fluid intake recommendations

**DOI:** 10.3389/fnut.2026.1923019

**Published:** 2026-10-13

**Authors:** Ge Song, Yue Chen, Jiaqi Li, Xinliang Pan, Hongtao Sui, Jianhao Chen, Yihsuan Lin, Yi Yan

**Affiliations:** 1 Department of Sport Biochemistry, School of Sport Science, Beijing Sport University, Beijing, China; 2 Beijing Research Institute of Sports Science, Beijing, China; 3 Changping No. 2 High School Education Group Yuzhi School, Beijing, China; 4 Xi'an University of Architecture and Technology, Xi'an, China; 5 Laboratory of Sports Stress and Adaptation of General Administration of Sport, Beijing, China

**Keywords:** athlete, fluid replacement, hydration, taekwondo, urine biomarker

## Abstract

**Background:**

Adequate hydration is crucial for athletic performance and health, particularly in adolescent athletes with distinct physiological demands. Comprehensive data on the daily and training-specific hydration status of populations like Chinese youth taekwondo athletes remain limited, hindering evidence-based strategy development.

**Objective:**

This study aimed to rigorously evaluate the daily and training session hydration status of taekwondo athletes in the Chinese youth team.

**Methods:**

This study evaluated the daily and training session hydration status of 58 Chinese youth taekwondo athletes (30 males, 28 females; age 17.7 ± 2.2 years) over a 7-day period. Assessment included 24-h rehydration behavior, daily fluid intake surveys, and objective hydration indices (urine osmolality, specific gravity, sweat ion concentrations).

**Results:**

A significant prevalence of suboptimal hydration, with 48.3% of athletes demonstrating persistent dehydration (defined as urine specific gravity >1.020 and urine osmolality ≥800 mOsm/kg) throughout the entire 7-day period. Median daily fluid intake for male athletes was 27.98 mL/kg BM, and for female athletes was 29.70 mL/kg BM; both values were below the daily fluid replacement volume of urine specific gravity ≤ 1.020 subjects for this athletic population. Training sessions significantly exacerbated dehydration, evidenced by elevated post-session urine specific gravity and osmolality of technical/tactical training session (*P*<*0.05*), alongside substantial median body mass loss rate of 2.51% for technical/tactical, 2.16% for physical fitness, and 1.97% for strength training sessions.

**Conclusion:**

This study identifies inadequate daily and training-induced hydration in Chinese youth taekwondo athletes. Based on the median values observed in euhydrated athletes in our cohort, daily fluid intake >30.35 mL/kg BM/day for males and >32.63 mL/kg BM/day for females on training days, alongside a training session intake of 1.87–2.78 mL/kg BM/h, may support maintenance of optimal hydration. However, total intake volume alone does not guarantee euhydration; beverage composition, timing of consumption, and pre-training hydration status are also important factors.

## 1 Introduction

Maintaining optimal hydration is crucial for physiological homeostasis, as both hyperhydration and hypohydration can lead to electrolyte imbalances and adverse health consequences (1). Optimal hydration status provides multiple benefits for athletic populations, including elevated testosterone levels (2), increased lean body mass (3), enhanced neuromuscular function (4), and improved athletic performance (5–7). Conversely, dehydration has been shown to impair multiple aspects of sports performance (8), with documented reductions in maximal oxygen uptake (4.62%) (9), strength (~2%) (10), explosive power (~3%) (10), endurance capacity (~10%) (10), anaerobic performance (11), and cognitive abilities including attention and memory (10). These findings underscore the importance of developing scientifically-based, individualized rehydration protocols that based on sweat and electrolyte loss in athletes (5). Given the substantial variations between sport events in terms of physical demands, equipment requirements, exercise intensity and duration, and environmental conditions, implementing sport-specific rehydration strategies becomes imperative. Such targeted approaches are necessary to maintain optimal hydration during training and competition, thereby supporting athletes' physiological function and competitive performance (12).

Taekwondo, an official Olympic combat sport, involves intense physical confrontation between two competitors. Training sessions and competitions typically occur in poorly ventilated indoor areas while athletes wear specialized uniforms that restrict heat exchange with the environment. The mandatory protective equipment, including gloves, chest protectors, elbow pads, and helmets–typically constructed from impermeable plastic materials–further impairs thermoregulatory capacity, consequently exacerbating fluid loss (13). Additionally, athletes frequently engage in rapid body mass reduction through deliberate dehydration in the pre-competition period to qualify for the lowest possible weight class within their range (14, 15). These practices collectively compromise both athletes' health and athletic performance. Given that taekwondo athletes demonstrate higher susceptibility to dehydration compared to other sports, establishing evidence-based rehydration protocols becomes particularly crucial. However, current research lacks sufficient experimental data regarding optimal fluid replacement volumes specifically for taekwondo athletes, hindering the development of scientifically validated hydration strategies.

Current methods for assessing hydration status include measurements of body mass, plasma indices (plasma osmolality and electrolyte concentrations), urine indices (volume, color, osmolality, and urine specific gravity) (16), and sweat indices (electrolytes such as sodium, chloride, and potassium). Recent studies have increasingly utilized urine and sweat indices as non-invasive indicators of hydration status (3, 16–18). This study investigated the hydration status of elite young taekwondo athletes in China through a 7-day assessment of: (a) 24-h fluid intake behavior, (b) urine and sweat indices, and (c) comprehensive hydration status evaluation. The findings provide scientifically validated data to inform the development of optimal rehydration strategies for taekwondo athletes while contributing to the establishment of more robust hydration assessment criteria.

## 2 Materials and methods

### 2.1 Study design and setting

This study employed a 7-day repeated-measures observational field study design to monitor hydration status, fluid intake, and sweat loss in youth taekwondo athletes during a regular training week. The study was conducted at the National Winter Sports Training and Research Base in Beijing, China, June 2023. The study period comprised 5 workdays (training days: Day 2–4, Day 6–7) and 2 weekend days (rest days: Day 1, Day 5). Throughout the observation period, athletes resided in the team dormitory and trained at the designated training facilities, with all meals provided by the team cafeteria under the supervision of team nutritionists. Environmental conditions (ambient temperature and relative humidity) were recorded throughout the 7-day period.

This study followed the Strengthening the Reporting of Observational Studies in Epidemiology (STROBE) guidelines (19) and STROBE checklist is provided as Supplementary material.

### 2.2 Participants

A total of 66 youth taekwondo athletes were recruited from the Chinese National Youth Training Team using whole-team sampling.

Inclusion criteria: (a) certified taekwondo athletes from the Chinese National Youth Training Team; (b) aged 14–24 years; (c) classified as national-class, first-class, or second-class and below by the General Administration of Sport of China; (d) in good physical health with no hypertension, cardiovascular diseases, or other chronic conditions; (e) in healthy mental condition with full capability to independently complete study questionnaires.

Exclusion criteria: (a) current use of medications affecting fluid balance or thermoregulation (e.g., diuretics, antihypertensives); (b) acute illness or injury at the time of enrollment; (c) inability to comply with the 7-day data collection protocol.

The study was approved by the Research Ethics Committee of Beijing Sport University (approval number: 2023103H) and adhered to the ethical principles of the Declaration of Helsinki. All participants and their legal guardians provided written informed consent prior to enrollment.

### 2.3 Sample size estimation

Given the non-normal distribution of hydration indices (e.g., urine specific gravity and urine osmolality) in this study, nonparametric statistical methods were employed. To ensure adequate statistical power, we conducted an *a priori* power analysis based on effect sizes reported in prior hydration study (20, 21). Using GPower 3.1.9.7 software (Heinrich-Heine-Universität Düsseldorf, Germany) with the Wilcoxon signed-rank test (matched pairs) model – appropriate for analyzing paired comparisons of non-normally distributed continuous/ordinal data – we performed an a priori power analysis given alpha, power, and effect size parameters. The analysis was configured with: normal parent distribution, Effect size *f* = 0.40, α error probability = 0.05 (two-tailed), and Power (1-β probability) = 0.8. The calculation yielded a required sample size of 54 matched pairs. To accommodate potential dropouts and biological variability in hydration responses, we enrolled 66 participants were enrolled.

### 2.4 Study procedure

The study was conducted for 7 consecutive days, and study protocol comprised three phases (Figure 1).

**Figure 1:**
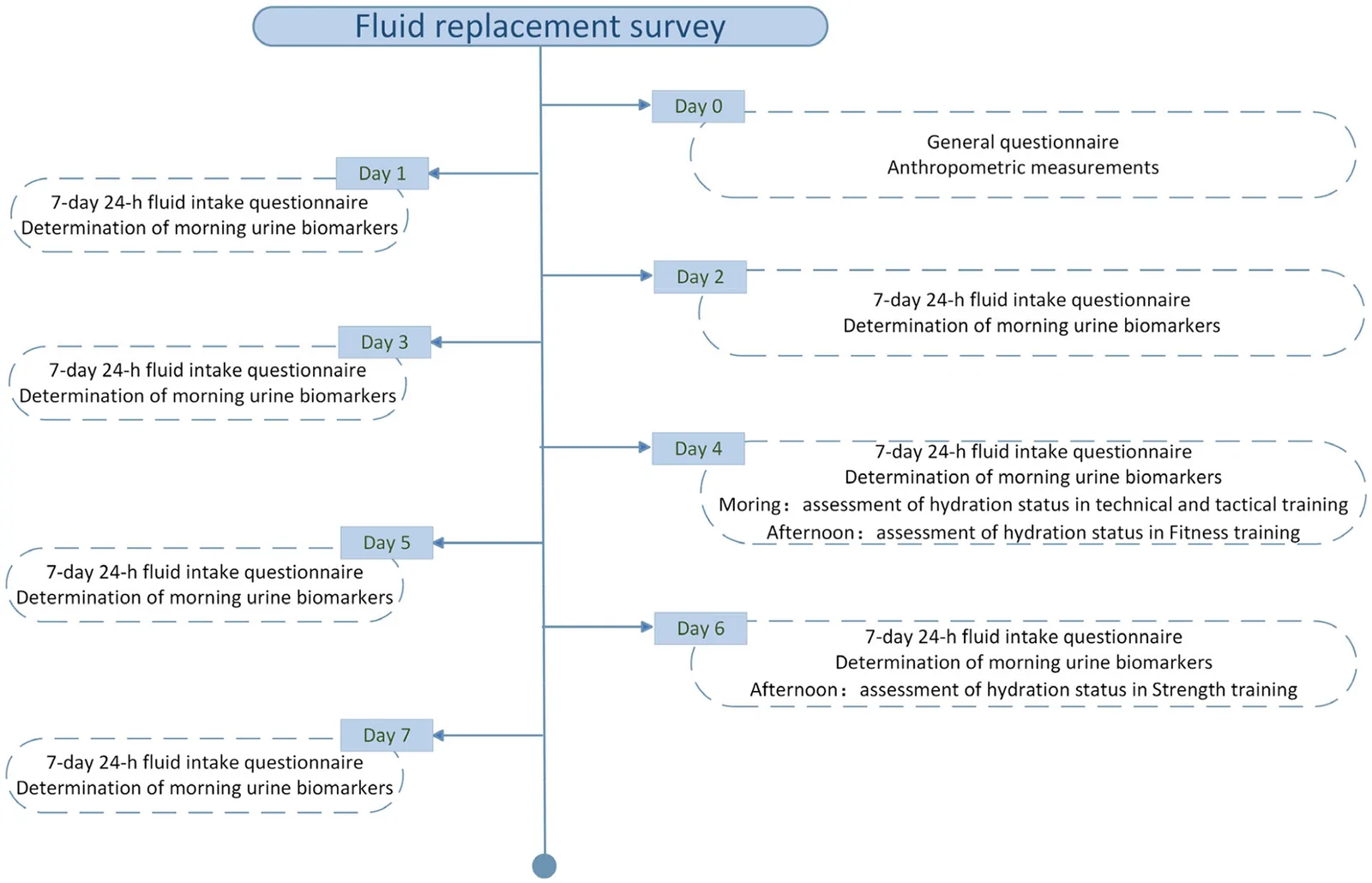
The study procedure.

(a) Pre-study preparation (Day 0): participants received standardized measuring cups, a 24-h fluid replacement record recording questionnaire, a general questionnaire (age, sex, training history, athletic level, education), and informed consent documents. Investigators and coaches thoroughly briefed athletes on recording procedures. Fasting body measurements (stature and body mass) were obtained after an 8-h overnight fast.
(b) 7-day observational monitoring (Day 1–7): athletes completed the daily Fluid Intake Record Sheet documenting consumption timing, beverage type, volume, location, and commercial product names (16). Investigators checked questionnaire completeness each next-day morning. First-morning mid-stream urine samples were collected each day for hydration assessment. Environmental conditions were recorded three times daily.
(c) Training session monitoring (Day 4 and Day 6): three training sessions were monitored: Day 4 morning (technical and tactical training session), Day 4 afternoon (physical training fitness session), and Day 6 afternoon (strength training session). Pre- and post-training assessments included body mass measurement, urine sampling, sweat patch collection, and continuous heart rate monitoring (see Section 2.9). Environmental conditions (Temperature and humidity of locations where athletes were located) were recorded to account for potential thermal influences on hydration status.

Of 66 recruited athletes, 58 athletes completed the full 7-day monitoring protocol (daily fluid intake questionnaires and first-morning urine samples) and were included in the daily hydration analysis. For training session analyses, 58 athletes completed the technical and tactical training session assessment, 59 completed the physical fitness training session assessment and 49 completed the strength training session assessment (fluid intake monitoring, sweat sample and pre- and post-exercise urine samples). These subsets overlapped but were not identical, as some athletes were unable to complete data collection (missing ≥1 urine samples or sweat sample). A detailed participant flow diagram is presented in Figure 2.

**Figure 2:**
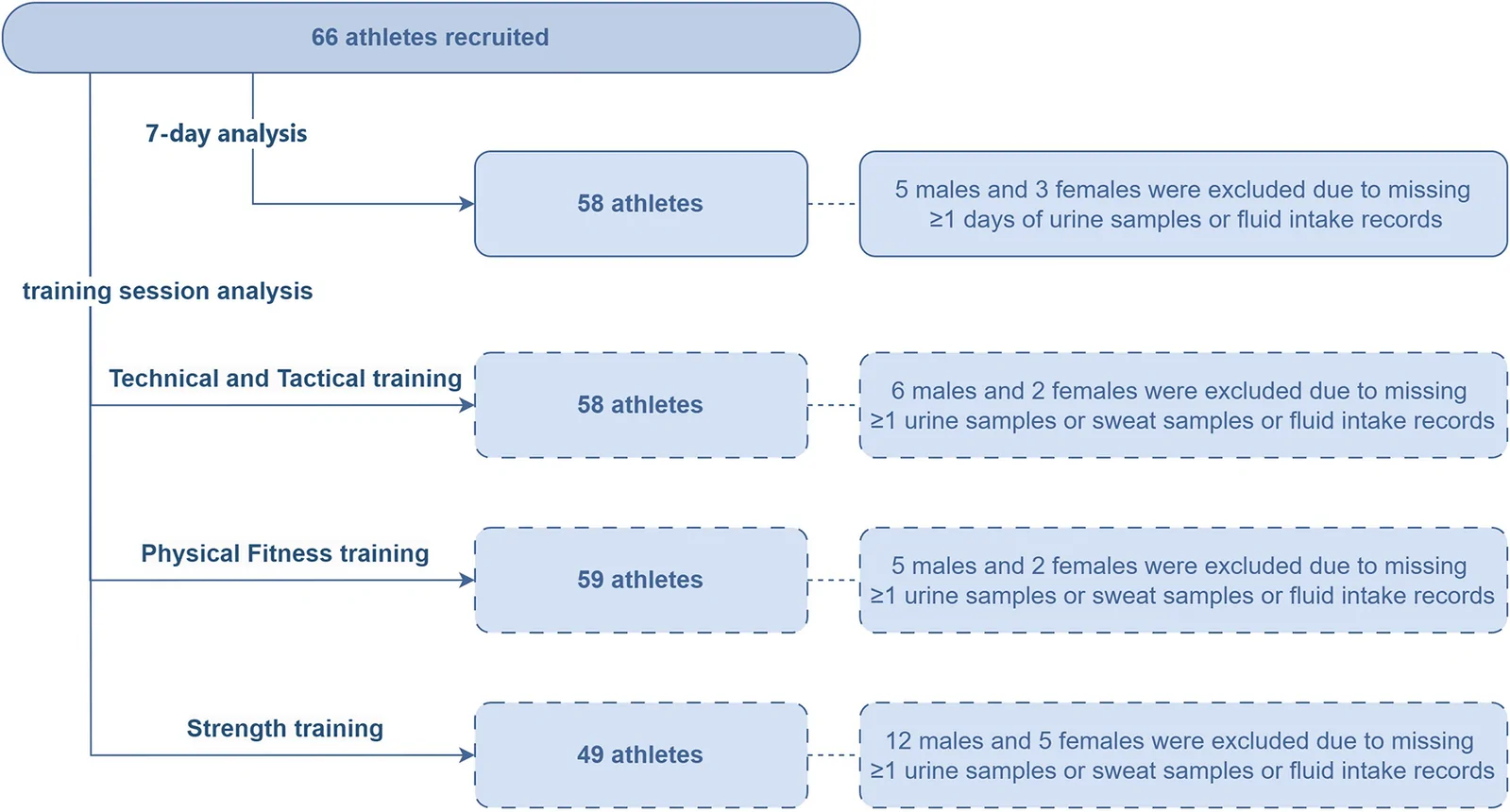
The participant flow diagram.

### 2.5 Training session monitoring

In order to gain an exhaustive understanding of the athlete's hydration status during training, we conducted a survey of hydration status and rehydration during technical and tactical training session, physical fitness training session, and strength training session along with a 7-day survey of hydration status and rehydration.

#### 2.5.1 Pre-training assessment

Fifteen minutes prior to each training session, athletes (fasted, no fluid intake) underwent: (a) body mass measurement wearing only dry undergarments; (b) pre-training mid-stream urine collection; (c) attachment of sweat absorbent patches on the dorsal forearm, upper back (scapular region), ventral midthigh, and calf after cleaning the skin with deionized water and drying with electrolyte-free gauze.

#### 2.5.2 During-training monitoring

During training, athletes used a standardized graduated cup (provided by investigators) for all fluid intake and self-recorded the volume, type, and timing of each drinking occasion. Heart rate (HR) were continuously recorded throughout each training session using the Firstbeat bodyguard 3 (chest straps) and team sports monitor system (Firstbeat, Finland). Investigators turned the HR receiver on and off simultaneously at the beginning and end of each training session.

#### 2.5.3 Post-training assessment

At the end of each training session, sweat patches were removed with electrolyte-free forceps, sealed in Ziploc bags, and stored at 4 °C. Post-training body mass was measured after towel-drying and changing into dry undergarments, and the first post-training urine samples were collected.

The content of the technical and tactical class was mainly kicking with different heights and routes, and the training duration was 177 min. The content of the physical fitness class was the comprehensive ability training such as frog jump and hill climb jump, and the training duration was 128 min. The content of the strength class was the resistance training such as squatting with different angles and body mass, and the grip push, and the training duration was 139 min.

### 2.6 Environmental conditions

Ambient temperature and relative humidity were recorded using a thermo-hygrometer (Zoglab, CN) with a measurement precision of ±0.1 °C for temperature and ±1% for relative humidity. Measurements were taken at the athletes' primary locations: dormitory (rest periods), training center (technical/tactical and strength training), gymnasium (physical fitness training), and outdoor slope/runway (specific training activities). Thermo-hygrometer placed at chest height (~1.5 m above floor level) in the center of each training area, away from direct sunlight or ventilation sources that could affect readings. Temperature and humidity were located were recorded at 10:00, 15:00 and 20:00 every day for 7 consecutive days. The temperature of the training and residence of the athletes ranged from 26.3 to 35.8 °C and the humidity ranged 21%−49% (Table 1).

**Table 1:** Temperature and humidity of the environment.

Date	The temperature and humidity								
	**10:00**			**15:00**			**20:00**		
	**Temperature (**°**C)**	**Humidity**	**Location**	**Temperature (**°**C)**	**Humidity**	**Location**	**Temperature (**°**C)**	**Humidity**	**Location**
D1	26.4	48%	Dormitory	26.5	49%	Dormitory	25.8	40%	Dormitory
D2	29.1	30%	Training center	26.3	48%	Gymnasium	26	37%	Dormitory
D3	28.9	37%	Training center	29.6	37%	Outdoor slope	26.5	38%	Training center
D4	34.4	21%	Training center	35.8	45%	Outdoor runway	26.3	33%	Dormitory
D5	26.4	39%	Dormitory	26.3	36%	Dormitory	26.4	34%	Dormitory
D6	29.8	36%	Training center	27.1	49%	Gymnasium	26.8	39%	Training center
D7	29.5	33%	Training center	27.4	48%	Training center	26.6	46%	Dormitory

### 2.7 Anthropometric measurements

Stature and body mass were measured by investigator with standard procedure. Athletes who fasted for 8 h were asked to wear light clothes and no shoes (22).


$$\begin{array}{l} \text{BMI}(\text{kg/m}2^{1})=\text{body mass}(\text{kg})/{\left[\text{stature}(\text{m})\right]}^{2}\\ \text{Body surface area}({\text{m}}^{2})={\text{stature}}^{0.425}(\text{kg})\times {\text{stature}}^{0.725}(\text{cm})\times 0.007184 \end{array}$$

### 2.8 Measurement of fluid replacement

Athletes were asked to complete the 24-h fluid replacement record recording questionnaire for 7 consecutive days (5 workdays and 2 weekends). The questionnaire was designed by the investigators and has high validity, as previously described (16). The type and volume of fluid replacement on each occasion was measured by a standardized cup provided by the investigator.

Total daily fluid replacement (mL/day) was defined as the sum of all beverages consumed between waking and sleeping, recorded using the 24-h fluid replacement recording questionnaire for 7-days.

Training-session fluid intake (mL) was defined as the volume consumed from 15 min before to 15 min after each training session, further subdivided into pre-training ( ≤ 15 min before onset), during-training, and post-training ( ≤ 15 min after cessation) periods.

Beverage types were categorized as: (a) plain water: plain water, mineral water, mineral water and purified water, etc.; (b) milk and milk products: fresh milk, powdered milk and yogurt, etc.; (c) sugary beverages: carbonated beverages, flavored beverages, etc.; (d) functional beverages: homemade or bottled sports beverages, energy beverages, electrolyte beverages, etc.; and (e) other beverages: tea, alcohol, etc., which do not belong to any of the above five types of beverages.

Body-mass-normalized fluid intake was calculated as mL/kg body mass/day for all analyses.

### 2.9 Urine indices

The middle part of morning urine was collected by the athletes themselves for 7 consecutive days and sent to the laboratory. Trained investigators recorded the urine samples and stored them in a 4 °C refrigerator. Testing was standardized when all samples were collected on the same day. Urine specific gravity was tested using the urine testing instrument (Clinitek status; Siemens, Germany), and urine osmolality was tested using the osmolality molar concentration meter (SMC 30C-1; Tianhe, CN).

Hypohydration was defined as urine specific gravity >1.020 and urine osmolality ≥800 mOsm/kg, consistent with established criteria used in athletic populations by the American College of Sports Medicine (ACSM) position stand (23) on exercise and fluid replacement and the National Athletic Trainers' Association (NATA) fluid replacement position statement (8).

### 2.10 Sweat collection and analysis

Before exercise, the subject's skin was shaved and cleaned with alcohol at the patch locations. The absorbent patch (HN-003; Hynaut, CN); size 10 cm^2^ was used to collect sweat from the following anatomical sites on the right side of the body: dorsal forearm, upper back (scapular region), ventral midthigh and calf. Immediately before patch application the skin was wiped dry with electrolyte-free gauze (7.5 × 7.5 in.; Hynaut, CN). Absorbent patch application before 5 min the onset of exercise. Patches were applied to the four anatomical sites in the same order for all subjects. At the end of the training session, the sweat patches were removed with electrolyte-free forceps, and sealed immediately in a Ziploc bag and stored at 4 °C. Regional sweat was uniformly separated for ion concentration analysis.

Regional Sweat ionized sodium (Na^+^), potassium (K^+^), and chloride (Cl^−^) concentrations were analyzed by ion-selective electrode (ISE) on an Easylyte analyzer (Medica corporation, USA).

Whole-body sweat ion concentration [Na^+^] was predicted from dorsal forearm regional sweat [Na^+^] using the validated and cross-validated regression equation of Baker et al. (24, 25):


$$\begin{array}{lll} Whole & - & \text{Body sweat}\left[\text{N}{\text{a}}^{+}\right]\\ = & 0.589\times dorsal\;forearm\;sweat\left[Na^{+}\right]+14.299 \end{array}$$

[Cl^−^] and [K^+^] were also determined according to the same methods as [Na^+^] listed above, but only descriptive data are shown.

### 2.11 Body mass loss analysis

Body mass was measured before and after each training session using a calibrated digital scale to the nearest 0.01 kg, with athletes wearing only dry undergarments after towel-drying sweat from the skin and hair. Urine output during training sessions was not measured.

To estimate total sweat loss corrected for fluid intake during training, the following formula was used (25):


$$\begin{array}{lll} Body\;mass\;loss\left(kg\right)\\ \; & = & pre-training\;body\;mass-post-training\;body\;mass\\ \; & + & fluid\;intake\;during\;training\\ Percentage\;of\;body\;mass\;loss\left(\%\right)\\ \; & = & Body\;mass\;loss/pre-\text{training body mass}\times 100\\ Body\;mass\;loss\;rate\left(g/min\right)\\ \; & = & Body\;mass\;loss/\text{training session duration} \end{array}$$

### 2.12 Energy expenditure in training lessons

Energy expenditure (kcal) was estimated from beat-to-beat heart rate (HR) and heart rate variability (HRV) data using the Firstbeat proprietary algorithm (Firstbeat, Finland), which incorporates a physiological model based on oxygen consumption (VO_2_) kinetics, excess post-exercise oxygen consumption (EPOC), HRV-derived respiration rate, and individual fitness characteristics. The chest strap electrocardiogram (ECG) sensor sampled RR-interval data at 1,000 Hz, and the EE estimation was performed using the built-in algorithm of the Firstbeat Sports analysis software without individual calibration (26).

### 2.13 Bias mitigation

Several measures were implemented to minimize potential sources of bias:

(a) Measurement bias: all body mass measurements were obtained by the same trained investigator using a single calibrated digital scale, with standardized pre-measurement conditions (fasting, dry undergarments, towel-dried). Urine samples were analyzed on the same day using the same instrument to eliminate inter-batch variability. Sweat patch placement followed a standardized order for all participants.
(b) Information bias: the 24-h fluid intake record relied on self-report; to mitigate recall and recording bias, investigators checked each daily record every second-day morning for completeness, cross-referencing entries with the standardized measuring cup graduations. Athletes were trained on proper recording techniques before the study.
(c) Selection bias: all eligible athletes from the National Youth Training Team were invited to participate, minimizing self-selection bias. The final analytical sample (*n* = 58) did not differ significantly from the enrolled sample (*n* = 66) in age, sex distribution, or athletic level.
(d) Confounding: environmental conditions (temperature, humidity) were recorded throughout the study to account for thermal influences on hydration status. Training intensity was objectively monitored via heart rate and energy expenditure. Dietary electrolyte intake was not controlled and is acknowledged as a potential confounder in the Limitations.

### 2.14 Data and statistical analysis

The data in this study are primarily given as median from quantile regression [M (QR)] to describe central tendency and dispersion, with partial data presented as mean ± standard deviation (M ± SD). For hydration status monitoring data across different training sessions, missing values were imputed using interpolation regression to obtain complete datasets. When data followed a normal distribution, one-way Analysis of Variance (ANOVA) was applied; for non-normally distributed data, we reported M (QR) and performed Kruskal–Wallis H tests followed by pairwise comparisons with Bonferroni correction. Between-group comparisons (including sex, training schedule, dehydration level, education background, training duration, athletic rank, and competition level) were conducted using Kruskal–Wallis tests with subsequent Bonferroni-adjusted pairwise comparisons.

## 3 Results

### 3.1 Characteristics of athletes

The study initially enrolled 66 elite taekwondo athletes representing China's highest competitive level in youth taekwondo. Among these participants, 58 athletes (30 males and 28 females) successfully completed the full 7-day monitoring protocol, with their data included in the final analysis. The cohort had a mean age of 17.7 years and an average training experience of 4.7 years, with detailed characteristics presented in Table 2. Athletes are competing in weight categories involving women-49 kg, women-57 kg, women-67 kg, women+67 kg, men-58 kg, men-68 kg, men-80 kg, men+80 kg.

**Table 2:** Demographic characteristics of athletes (M ± SD).

**Demographic group**	Age	Stature (m)	Body mass (kg)	BMI (kg/m^2^)	Body surface area (m^2^)	Frequency (percentage) [*n* (%)]
Total	17.7 ± 2.2	1.83 ± 0.07	66.94 ± 10.67	19.82 ± 2.11	1.87 ± 0.17	58 (100)
Gender						
Male	17.4 ± 1.9	1.87 ± 0.06	70.90 ± 11.11	20.36 ± 2.26	1.94 ± 0.17	30 (52)
Female	18.0 ± 2.4	1.80 ± 0.06	62.69 ± 8.48	19.25 ± 1.82	1.80 ± 0.14	28 (48)
Training years						
≤ 3.0	16.1 ± 1.4	1.83 ± 0.07	64.93 ± 11.87	19.36 ± 2.31	1.84 ± 0.18	21 (36)
3.1–5.0	17.6 ± 1.4	1.82 ± 0.06	65.76 ± 8.41	19.68 ± 1.72	1.85 ± 0.14	17 (29)
>5.0	19.5 ± 1.98	1.85 ± 0.08	70.05 ± 10.86	20.43 ± 2.17	1.92 ± 0.18	20 (35)
Athletic grades						
National-class athlete	20.7 ± 2.5	1.83 ± 0.07	66.80 ± 6.60	19.89 ± 1.73	1.87 ± 0.11	7 (12)
First-class athlete	18.2 ± 1.4	1.84 ± 0.07	67.3 ± 11.14	19.95 ± 2.22	1.88 ± 0.18	25 (43)
Second-class athlete and below	16.4 ± 1.7	1.83 ± 0.07	66.6 ± 11.4	19.69 ± 2.18	1.87 ± 0.18	26 (45)
Education level						
Undergraduate	23.0 ± 2.9	1.88 ± 0.05	69.88 ± 8.59	19.83 ± 1.40	1.94 ± 0.14	2 (3)
Senior school	18.4 ± 1.7	1.85 ± 0.07	68.82 ± 11.27	20.18 ± 2.27	1.90 ± 0.18	36 (62)
Junior school	15.9 ± 1.2	1.81 ± 0.07	63.26 ± 9.07	19.18 ± 1.78	1.81 ± 0.15	20 (35)

### 3.2 Measurement of 7 days fluid replacement

During the 7-day monitoring period, fluid intake was recorded via a 24-h recall questionnaire administered to each athlete. The median daily total fluid intake (QR) over the entire 7-day period was 29.0 (22.0–34.0) mL/kg BM (1,861 mL), with a median of 6.4 (5.1–7.6) drinking occasions per day and a median volume of 290 (260–336) mL per drinking episode. On workdays specifically, the median daily fluid intake was 34.1 (23.8–39.8) mL/kg BM (2,145 mL), with a median of 7.1 (5.4–8.7) drinking occasions per day and a median volume of 300 (269–359) mL per episode. A comparison of rehydration volumes between workdays and weekends was conducted and found that rehydration volumes were much higher on all workdays, regardless of gender, training years, athletic grades and education level, except of undergraduate subjects (*P* < 0.05; Figure 3). Evaluation of athletes' total fluid intake across 7 days are provided in Supplemental Table 1.

**Figure 3:**
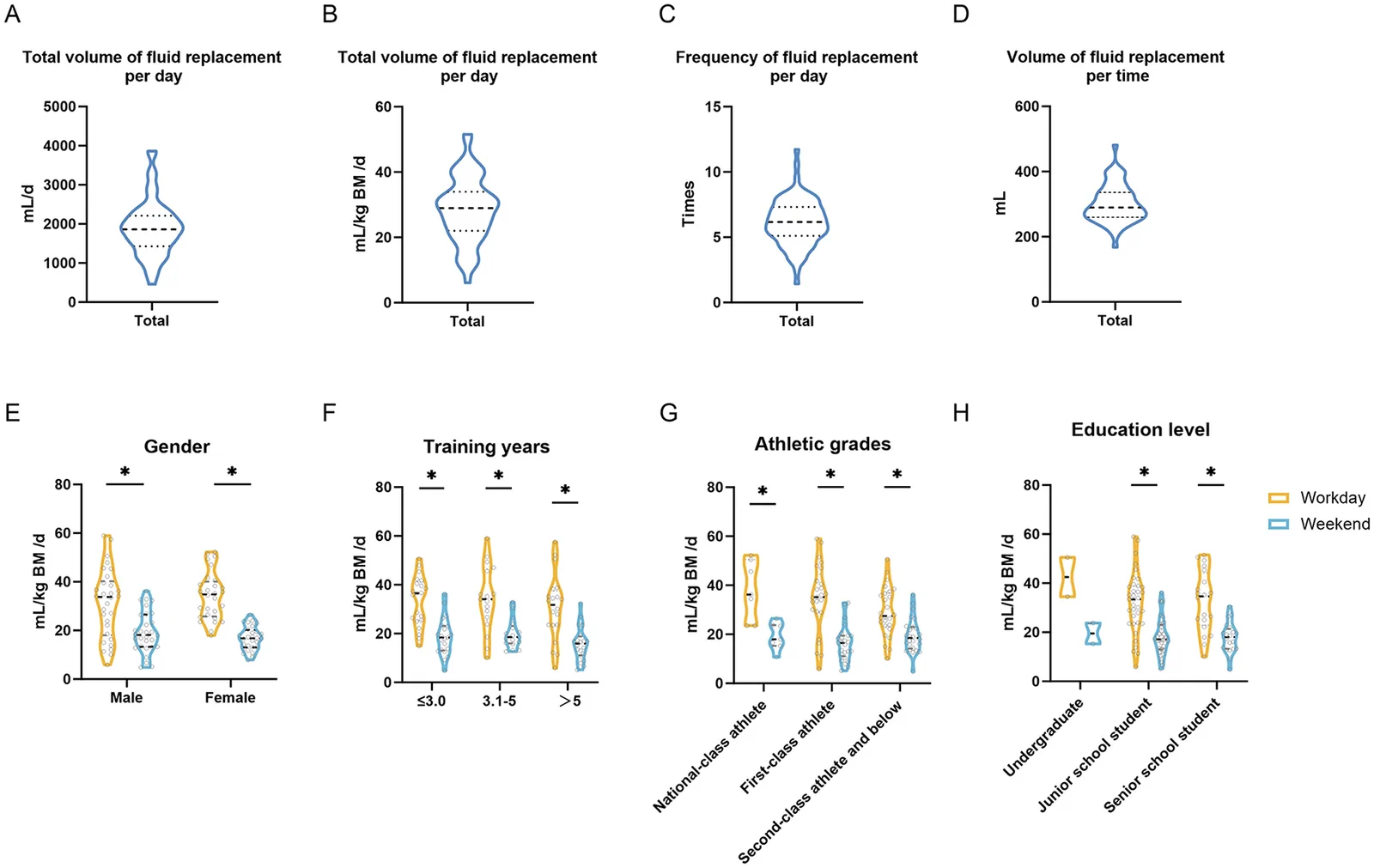
Athletes' fluid replacement behavior over 7 days. **(A, B)** Total volume of fluid replacement per day; **(C)** The frequency of fluid replacement per day; **(D)** The volume of fluid replacement per time; **(E–H)** Workday and weekend fluid replacement for different genders, training years, athletic levels, and education levels. *P < 0.05 compared to workday.

### 3.3 Measurement of urine indices

During the 7-day study period, we collected a total of 406 first-morning urine samples from 58 athletes, and the 7-day hydration status of the athletes showed a skewed distribution. Both urine specific gravity and urine osmolality are important indicators for evaluating hydration status. According to the urine specific gravity >1.020 for hypohydrated, 85.5% (347) of the subject were hypohydrated and when according to the urine osmolality ≥800 mOsm/kg, 88.2% (358) of the participant were hypohydrated. In this study, the percentage of athletes with urine specific gravity >1.020 and urine osmolality ≥800 mOsm/kg was counted, approximately 48.3% of the respondents were hypohydrated for 7 days. Among the 58 athletes, only one athletes maintained euhydration (urine specific gravity ≤ 1.020 and urine osmolality < 800 mOsm/kg) on all 7 monitoring days. The percentage of females who were hypohydrated on a 7-days average was slightly higher than that of males (50 vs. 46.6%), and the percentage of 7-days hypohydrated in athletes with >5 training years was lower than others (Figure 4).

**Figure 4:**
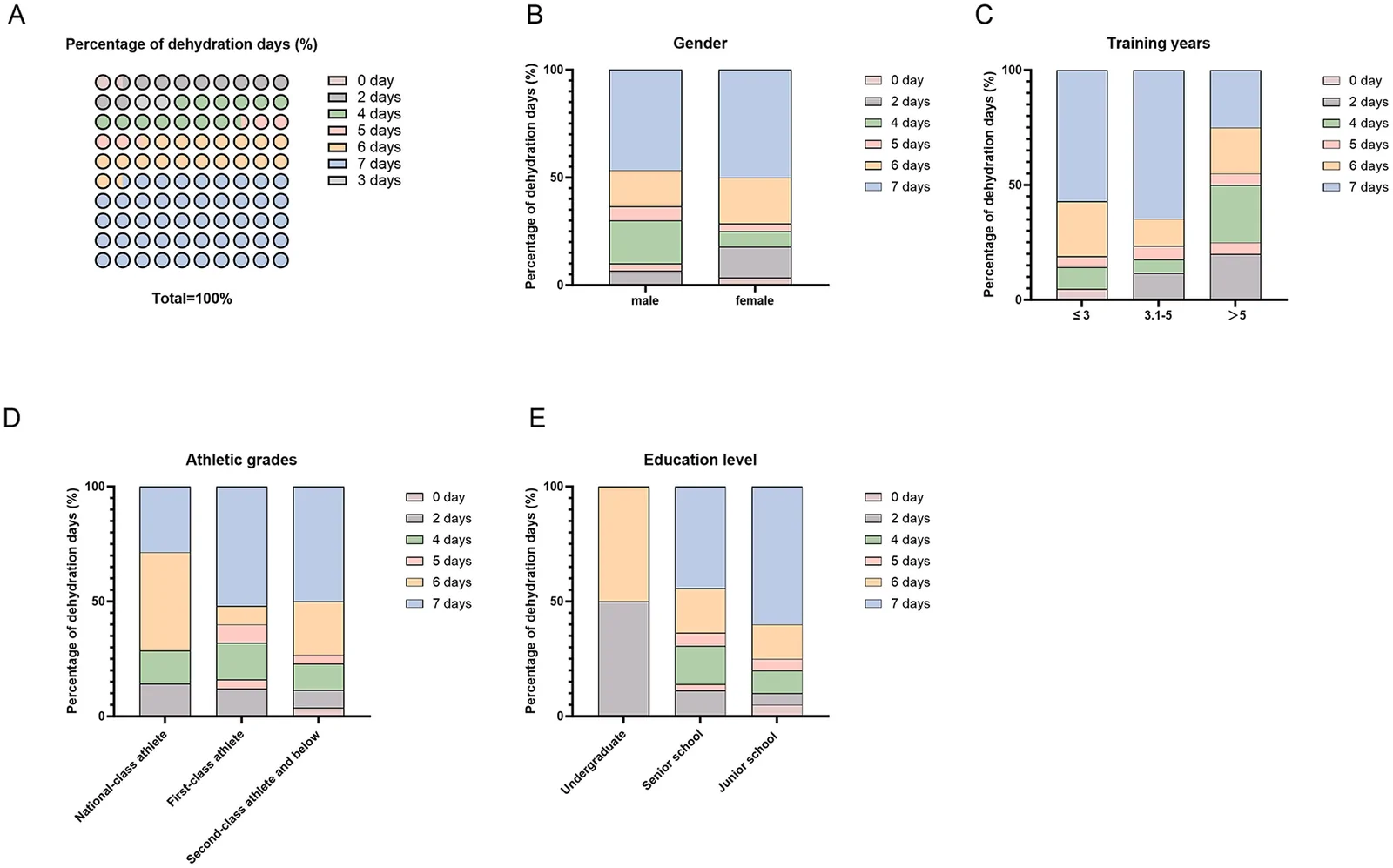
Days of dehydration in athletes when urine specific gravity >1.020 and urine osmolality ≥800 mOsm/kg. **(A)** The percentage of dehydration for different days; **(B–E)** The percentage of dehydration on different days across genders, training years, athletic levels, education levels.

### 3.4 Measurement of hydration of training lessons

In this study, we investigated the total volume of fluid replacement in three training sessions of the athletes. Table 3 showed the demographic characteristics of athletes and intensity of training sessions. The total volume of fluid replacement included the volume 15 min before, during and 15 min after the training session. The median volume of fluid replacement was 14.20, 10.24 and 10.24 mL/kg BM for technical and tactical training sessions, physical fitness and strength training sessions (Figure 5, Supplementary Table 2). We found that rehydration was significantly higher in the technical and tactical training sessions than in the physical fitness and strength training sessions (*P* < 0.05). Fluid intake during the 15-min pre- and 15-min post-training periods accounted for only a small proportion of total session fluid intake, with the majority consumed during training itself. Regardless of the training session, the preferred type of rehydration for the athletes was plain water, followed by sports drinks and sugary drinks, especially in the technical and tactical training sessions, where 94.8% of the athletes chose plain water for rehydration (Figure 5). Evaluation of athletes' fluid intake across training periods are provided in Supplemental Table 3.

**Table 3:** Demographic characteristics and intensity of training for athletes in different training sessions (M ± SD).

Indices	Technical and tactical training (***n*** = 58)		Physical fitness training (***n*** = 59)		Strength training (***n*** = 49)	
	Male (***n*** = **29)**	**Female (*****n*** = **29)**	**Male (*****n*** = **30)**	**Female (*****n*** = **29)**	**Male (*****n*** = **23)**	**Female (*****n*** = **26)**
Age	17.24 ± 1.88	17.92 ± 2.24	17.51 ± 1.88	17.99 ± 2.37	17.51 ± 1.88	18.03 ± 2.18
Stature (m)	1.88 ± 0.66	1.80 ± 0.07	1.87 ± 0.07	1.80 ± 0.06	1.86 ± 0.06	1.81 ± 0.06
Body mass (kg)	68.83 ± 14.90	62.55 ± 8.92	71.64 ± 11.47	63.13 ± 8.66	70.09 ± 8.76	63.84 ± 8.54
BMI (kg/m^2^)	20.07 ± 2.46	19.19 ± 1.84	20.52 ± 2.32	19.35 ± 1.86	20.31 ± 1.99	19.35 ± 1.79
Body surface area (m^2^)	1.96 ± 0.17	1.80 ± 0.15	1.95 ± 0.173	1.81 ± 0.15	1.93 ± 0.14	1.82 ± 0.14
Heart rate (time/min)	118.67 ± 11.95	123.75 ± 10.04	132.63 ± 11.31	131.78 ± 7.79	103.67 ± 7.20	108.50 ± 15.63
Energy expenditure (kcal)	1,068.56 ± 301.80	778.63 ± 210.48	820.13 ± 191.30	606.33 ± 120.45	444.33 ± 237.95	371.00 ± 146.32

**Figure 5:**
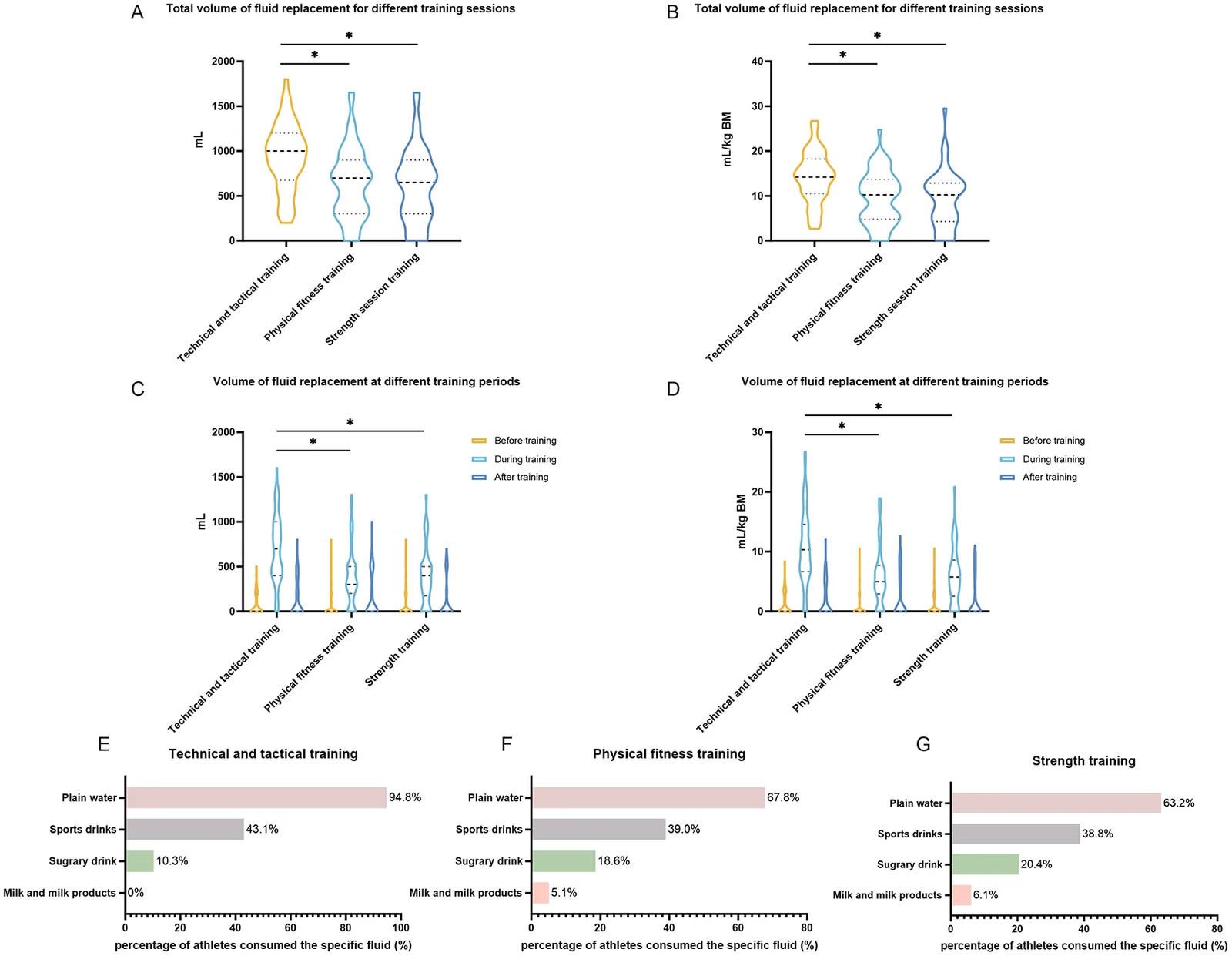
Fluid replacement for three training sessions. **(A, B)** Total volume of fluid replacement for different training sessions; **(C, D)** The volume of fluid replacement before, during or after training; **(E–G)** Types of fluid replacement in technical and tactical training session, physical fitness training session, strength training session. *P < 0.05 compared to technical and tactical training.

The athletes were hypohydrated prior to all three training sessions, and both urine specific gravity and osmolality increased significantly (*P* < 0.05) after the technical and tactical sessions, exacerbating the dehydration. Although urine specific gravity and osmolality did not significantly increase after the physical fitness and strength training sessions, the athletes were hypohydrated and all showed body mass loss after the training sessions (Figure 6, Table 4). Between-session comparisons of urine specific gravity and osmolality (pre- and post-training) are provided in Supplemental Figure 1.

**Figure 6:**
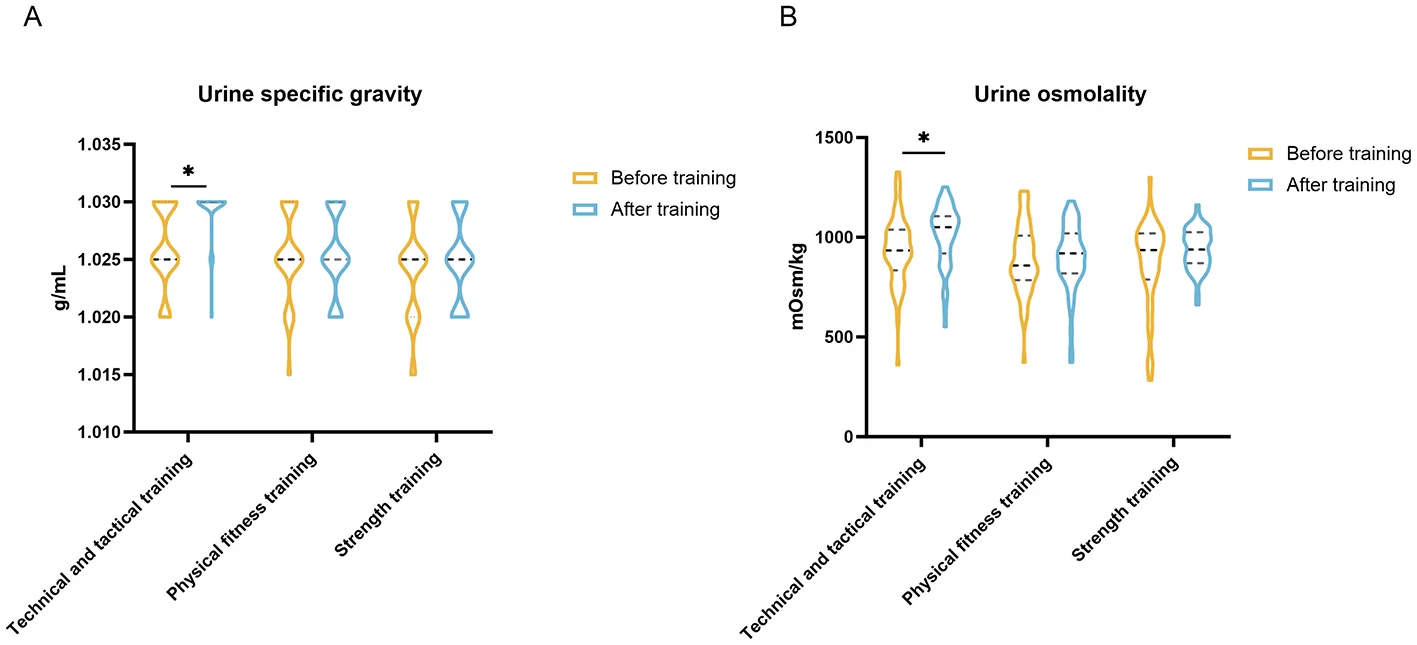
Urine indices before and after three training sessions. **(A)** Urine specific gravity; **(B)** Urine osmolality. *P < 0.05 compared to before training.

**Table 4:** Body mass, sweat and ionic loss of athletes in training sessions [M (QR)].

**Indices**			Technical and tactical training	Physical fitness training	Strength training
Body mass loss (kg)			−1.75 (0.76)	−1.45 (0.63)^*^	−1.25 (0.80)^*^
Percentage of body mass loss (%)			−2.51 (1.25)	−2.16 (0.82)^*^	−1.97 (0.87)^*^
Body mass loss rate (g/min)			−9.89 (4.31)	−11.33 (4.92)	−8.99 (5.76)^#^
Sweat ion concentration (mmol/L)	Whole body	Na^+^	40.04 (15.08)	44.46 (17.26)	33.32 (8.89)
		K^+^	18.04 (1.50)	18.43 (1.73)	19.35 (1.76)
		Cl^−^	34.18 (13.55)	38.33 (16.37)	27.79 (10.78)
	Dorsal forearm	Na^+^	43.70 (25.60)	51.20 (29.30)	32.30 (15.10)
		K^+^	6.35 (2.57)	7.02 (2.94)	8.58 (2.98)
		Cl^−^	33.75 (23.00)	40.80 (27.80)	22.90 (18.31)
	Upper back	Na^+^	46.60 (23.71)	60.30 (35.20)	38.70 (22.90)
		K^+^	5.00 (1.68)	5.05 (1.77)	6.59 (2.75)
		Cl^−^	36.65 (26.30)	28.10 (51.79)	28.90 (17.80)
	Ventral midthigh	Na^+^	40.50 (18.08)	42.90 (23.40)	38.37 (24.49)
		K^+^	6.70 (3.68)	7.44 (2.57)	8.18 (2.75)
		Cl^−^	29.90 (16.22)	33.90 (21.10)	24.60 (13.58)
	Calf	Na^+^	44.15 (20.97)	46.20 (31.68)	35.27 (17.14)
		K^+^	7.85 (2.42)	8.47 (2.34)	9.19 (4.19)
		Cl^−^	30.50 (19.73)	33.02 (29.42)	25.91 (16.77)

^*^P < 0.05 compared to technical and tactical training, ^#^P < 0.05 compared to physical fitness training.

The median body mass loss and percentage change following each training session type are presented in Table 4. The technical and tactical training session resulted in the most body mass loss (1.75 kg; 2.51% of body mass), which was higher than both the physical fitness training session (1.45 kg; 2.16%) and the strength training session (1.25 kg; 1.97%). When normalized to session duration, sweat rate (body mass loss rate) during the physical fitness training session (11.33 g/min) was higher than strength training session (8.99 g/min).

Whole-body sweat sodium, chloride, and potassium concentrations are also shown in Table 4. Median sweat sodium concentrations were 40.04 mmol/L for technical and tactical training, 44.46 mmol/L for physical fitness training, and 33.32 mmol/L for strength training. Corresponding median sweat chloride concentrations were 34.18, 38.33, and 27.79 mmol/L, respectively. Sweat potassium concentrations were similar across sessions, with median values ranging from 18.04 to 19.35 mmol/L.

### 3.5 Appropriate rehydration regimen for taekwondo athletes

Based on a urine specific gravity of ≤ 1.020 as optimal hydration, the median fluid replacement volume observed in athletes meeting this criterion on training days was 30.35 mL/kg BM for males and 32.63 mL/kg BM for females per day. The median fluid replacement volume of the athletes meeting this criterion on weekend was 20.16 mL/kg BM for males and 12.25 mL/kg BM for females per day (Table 5).

**Table 5:** Fluid replacement in athletes maintaining euhydration (urine specific gravity ≤ 1.020) by gender and training [M (QR)].

Day type	Gender	Fluid replacement volume (mL/kg BM)
Workday	Male	30.35 (10.56)
	Female	32.63 (13.67)
Weekend	Male	20.16 (13.67)
	Female	12.25 (7.32)

The American College of Sports Medicine recommends that athletes limit dehydration to less than 2% of body mass during training and competition. The median rehydration volume of athletes meeting this standard in technical and tactical training session was 2.78 mL/kg BM/h for males, and 2.22 mL/kg BM/h for females during the training; the median rehydration volume in physical fitness session was 2.45 mL/kg BM/h for males and 2.86 mL/kg BM/h for females during the training; and the median rehydration volume in strength session was 2.03 mL/kg BM/h for males and 1.87 mL/kg BM/h for females during the training. The relevant fluid replacement volumes are shown in Table 6.

**Table 6:** Fluid replacement in athletes maintaining euhydration (body mass loss < 2%) during training for different genders and training sessions M (QR).

Training sessions	Gender	Fluid replacement volume (mL/kg BM)	Fluid replacement volume per hour (mL/kg BM/h)
Technical and tactical training	Male	10.15 (7.52)	2.78 (3.70)
	Female	10.60 (7.65)	2.22 (0.00)
Physical fitness training	Male	4.97 (6.95)	2.45 (3.19)
	Female	6.43 (6.81)	2.86 (1.70)
Strength training	Male	5.77 (6.08)	2.03 (0.98)
	Female	6.63 (4.72)	1.87 (1.84)

## 4 Discussion

This study represents the first systematic investigation of hydration status among taekwondo athletes in China, employing comprehensive monitoring of rehydration patterns and physiological hydration markers. Through detailed assessment of both urinary and sweat indices, we identified several key findings regarding athletes' hydration status. The 7-day observation period revealed that most taekwondo athletes maintained a chronic state of dehydration and as evidenced by elevated urine specific gravity and osmolality that were significantly higher on training days compared to rest days, despite increased fluid intake during training periods. Notably, pre-training urine indices assessments indicated that most athletes began their sessions already in a hypohydrated state, with this condition significantly exacerbating following the technical and tactical training session. This suggesting these high-intensity sessions may impose particularly severe fluid balance challenges.

Fluid replacement plays a critical role in maintaining proper hydration status among athletes. Our findings demonstrate that the median values of fluid replacement of taekwondo athletes on training days (2,145 mL) are higher than what is reported in both the general Chinese population and physically active individuals. This fluid replacement exceeds the recommended amount of the Dietary Guidelines for Chinese Residents (1,700 mL for males and 1,500 mL for females) (27), surpasses the average consumption of Chinese male college students (1,134 mL) (28), and is higher than the median of fluid intake among Chinese adults (1,679 mL for males and 1,370 mL for females) (29) and the median consumption of regularly exercising male college students (1,789 mL) (16). Despite these elevated intake levels, biomarker analysis revealed persistent dehydration of taekwondo athletes, as indicated by elevated urine specific gravity and osmolality measurements. This suggests that taekwondo athletes have substantially greater fluid requirements than both general population and regular sports population, highlighting the need for sport-specific hydration guidelines for them. Current understanding of athletic hydration needs remains largely derived from endurance sports (30, 31), with limited data available for combat sports like taekwondo.

Using established clinical thresholds (urine osmolality ≤ 500 mOsm/kg) for optimal hydration (32) and >800 mOsm/kg indicating dehydration (28, 33), our data show that approximately 70% of taekwondo athletes maintained hypohydrated states during the study period. Athletes frequently experience dehydration, with a study of 28 college soccer players showing urine specific gravity >1.020 in 80% of measurements (12/15 days) (34). In our study, the percentage of females who were hypohydrated on a 7-day average was slightly higher than that of males, possibly due to gender-specific physiological factors including menstrual cycle effects. These findings collectively underscore the importance of developing tailored hydration strategies that account for both sport-specific demands and individual characteristics such as gender. The development of such guidelines should consider not only performance optimization but also long-term health outcomes, as chronic dehydration has been associated with increased risks of chronic diseases such as urolithiasis and cardiovascular disease, etc. (35, 36).

Heavy exercise and unscientific rehydration practices predispose to varying degrees of dehydration, but rehydration before, during, and after exercise prevents dehydration from affecting exercise capacity and reduces the risk of convulsions and syncope (37). Appropriate and individualized rehydration strategies should take into account pre-exercise hydration status as well as fluid and electrolyte requirements before, during, and after exercise (12). We therefore investigated the amount of fluid rehydration in athletes' training sessions. We found that most of the athletes do not rehydrate before and after exercise, thus contributing to dehydration in training sessions. Our results were similar to the study of Belfort et al. (13) that most of the athletes were already in a state of mild dehydration (urine specific gravity >1.020) before the training lessons.

Plain water was the most preferred type of rehydration for the athletes in our study, and although plain water can make up for some of the lost fluids, the athletes in this study had significant electrolyte losses due to their training day training duration of up to 5 h.

Sweat electrolyte composition plays an important role in exercise performance and thermoregulation (38, 39). The large loss of sweat and electrolytes during exercise is a contributing factor to heat stroke, heat cramps, heat exhaustion, and increases cardiovascular stress and renal dysfunction (20). During strenuous exercise, sodium ions are lost in large quantities along with sweat, leading to hyponatremia with reduced sodium ion concentration in the body, which in turn affects muscle contraction and even convulsions (40). Chloride ions and sodium ions are important ions for maintaining fluid balance, and together they control the flow of water in and out of cells by regulating the flow of positive and negative charges in ion channel cells (41, 42). In our study, whole-body sweat sodium and chloride concentrations showed numerical differences across training session types, though these data were presented descriptively without formal between-group statistical comparisons. These numerical trends may reflect differences in exercise intensity and ambient temperature. Sodium concentrations exceeded 40 mmol/L in both the technical and tactical session (40.04 mmol/L; 34.4 °C) and the physical fitness session (44.46 mmol/L; 35.8 °C) compared with 33.32 mmol/L in the cooler strength training session (27.1 °C). It is suggested that exercise in hot and humid environments should be more focused on electrolyte supplementation. It was found that reduced potassium ion levels in athletes were associated with the onset of fatigue after high-intensity training, suggesting that potassium ions should be supplemented promptly (43). Whereas drinking drinks containing carbohydrates and electrolytes before and during exercise can delay the onset of muscle cramps, thus prolonging the duration of exercise (40), supplementation with electrolyte-containing beverages during exercise is more effective than supplementation with plain water in reducing body mass loss.

The American College of Sports Medicine recommends that athletes limit dehydration to less than 2% of body mass during training and competition (23), and there have been many previous studies in many sports that have shown that loss of body fluids above 2% of body mass leads to a decrease in athletic performance (44–46). In this study, it was found that 81.0% in technical and tactical training session, 62.7% in physical training session, and 46.9% in strength training session lost more than 2% of their body mass. This indicated the amount of rehydration in three sessions was insufficient, especially in technical and tactical training session. Technical and tactical skills directly influence competitive performance in taekwondo, as elite athletes must skillfully combine various kicking and punching techniques to maximize scoring opportunities (47, 48). Consequently, technical and tactical training typically constitutes the largest portion of the training schedule among the taekwondo athletes in our study. This study also found that the dehydration of athletes was more pronounced after the technical and tactical training, which suggests that coaches and dietitians as well as the athletes themselves need to pay more attention to the hydration status of the technical and tactical training. The results of a survey on the hydration status of elite soccer players during training showed that after the training session equivalent to a level of dehydration of 1.37 ± 0.54% of the pre-training body mass. Our survey reveal a higher body mass loss than those of soccer players, with higher the urine osmolality and specific gravity (49). This indicates that the indicators should be integrated when assessing dehydration in athletes. A previous study examining cognitive function and Taekwondo-specific kicking performance across different hydration states demonstrated that athletes in a euhydrated state exhibited faster reaction speed, higher kicking success rates, and lower post-exercise heart rates compared to those in hypohydrated or severely hypohydrated conditions (18). Given that optimal hydration is closely linked to reaction speed and technical performance–and despite the high prevalence (89%) of rapid weight loss via dehydration among combat sport athletes during competition periods (20)–maintaining euhydration remains critically important for taekwondo athletes during both training and competition (18).

There are some drawbacks in this study. The sweat biomarker calculations relied on regional sampling (dorsal forearm, upper back, ventral midthigh and calf) combined with estimation formulas, which may not fully capture whole-body electrolyte losses due to known regional variation in sweat composition (50, 51). While direct whole-body washdown techniques exist, they require controlled laboratory settings and immediate post-exercise processing that are impractical for field studies with athletes (51). Similarly, standardized absorbent patch protocols, though more feasible than washdown, still demand careful standardization of patch size, placement, and timing across body regions (50, 52). These methodological constraints highlight an urgent need for the development of portable, non-invasive direct sweat collection methods that can provide real-time, whole-body electrolyte measurements in training environments (53). While the regression equations for predicting whole-body sweat ion concentrations from regional samples of Baker et al. (24, 25) were validated in adults, their application to a youth cohort requires caution given potential age-related differences in sweat gland function and electrolyte composition.

In addition, fluid intake behaviors were assessed via a self-reported 24-h recall questionnaire, which–while commonly used in field-based hydration research–is susceptible to recall bias and social desirability bias, potentially leading to misreporting of actual fluid consumption. Also, the questionnaire quantified fluid replacement exclusively through beverage consumption, the omission of water derived from food and metabolic water inherently underestimates the athletes' true total daily water intake. Furthermore, dietary electrolyte intake and the composition of consumed foods and beverages were not systematically assessed, which represent key confounding factors that may influence both sweat electrolyte concentrations and hydration status. Given that electrolyte replacement affects body hydration status (5, 54), and the present study quantified ion losses and fluid replacement separately, the joint effect of these two factors on hydration status warrants further investigation.

The environment and temperature are the key factors affecting the regulation of body fluids (55, 56), and since the test was conducted during the summer, when temperatures are higher relative to the winter, and body fluids are more easily lost through sweat, additional data on the hydration status of taekwondo athletes during the winter months is also needed in the future. Furthermore, environmental temperatures varied across training sessions (ranging from 27.1 °C to 35.8 °C, Table 1), which introduces a confounding variable when comparing body mass loss between training modalities. While this variability reflects real-world training conditions, it limits the ability to attribute differences in body mass loss solely to exercise intensity or training type. Future studies employing controlled environmental conditions or statistical adjustment for ambient temperature would help disentangle the respective contributions of exercise modality and environmental factors to fluid loss.

Additionally, because urine output during training sessions was not measured, sweat loss estimates calculated from body mass change and fluid intake may systematically underestimate actual total fluid loss during exercise. Unmonitored urinary excretion during multi-hour training sessions introduces a source of systematic error into body mass loss rate calculations (23, 57), which in turn affects the precision of our session-specific fluid intake guidelines. Consequently, the body mass loss values reported in our study should be interpreted as lower-bound estimates, and the corresponding fluid intake recommendations should be considered conservative. Future studies incorporating real-time urine collection during training would provide more precise quantification of this confounding variable.

We also acknowledge that the use of first-morning urine samples with a urine specific gravity >1.020 threshold may overestimate the true prevalence of dehydration. As demonstrated by Armstrong et al. (58) in 2010, first-morning urine samples are more concentrated than 24-h collections in approximately 85% of cases due to overnight fluid restriction and reduced glomerular filtration during sleep. While we applied dual biomarker classification (urine specific gravity *and* osmolality) to improve diagnostic specificity, these two measures share strong collinearity and do not fully correct for the circadian baseline elevation inherent to morning samples. Consequently, the prevalence estimates of hypohydration reported in this study should be interpreted with caution, as they may represent an upper bound rather than the true population prevalence. Nevertheless, the overall conclusion that suboptimal hydration is common in this athletic population remains supported by multiple convergent findings, including elevated post-training urine biomarkers and substantial body mass loss during training sessions.

We must acknowledge that the omission of a non-athlete control group or athletes from a sport with lower fluid loss to contextualize the dehydration levels in our study constitutes a limitation, although this design choice serves to better elucidate athletes' hydration status. It is our intention to address this methodological constraint in subsequent research endeavors by implementing more comprehensive experimental designs.

## 5 Conclusions

The daily hydration status of Chinese youth taekwondo athletes was suboptimal, with most athletes were in a hypohydrated state throughout the 7-day monitoring period. The amount of rehydration in technical and tactical training session, physical fitness training session, and strength training session of taekwondo athletes was insufficient. Before the training sessions, the survey respondents were in a hypohydrated state, and the degree of dehydration increased at the end of the training sessions. Based on the median values observed in euhydrated athletes in our cohort, daily fluid intake of 30.35 mL/kg BM for males and 32.63 mL/kg BM for females per day on training days, alongside a session intake of 1.87 - 2.78 mL/kg BM/h fluids, may support maintenance of optimal hydration. However, total intake volume alone does not guarantee euhydration; beverage composition, timing of consumption, and pre-training hydration status are also important factors. Hydration status awareness and behaviors of taekwondo athletes and coaches need to be improved. Targeted rehydration education and the promotion of healthier body mass management strategies are warranted for this athletic population.

## Data Availability

The original contributions presented in the study are included in the article/Supplementary material, further inquiries can be directed to the corresponding author.
