# Supplementary material for: Optimizing hydration in Chinese youth taekwondo athletes: evidence-based daily and training session-specific fluid intake recommendations
Source: Front Nutr. 2026 Oct 13;13:1923019. doi: 10.3389/fnut.2026.1923019 (PMC13710252; doi:10.3389/fnut.2026.1923019)
Supplement: Supplementary file 1 [file Supplementary_File_1.pdf]

STROBE Statement—checklist of items that should be included in reports of observational studies

	Item No.	Recommendation	Page No.	Relevant text from manuscript
**Title and abstract**	1	(*a*) Indicate the study's design with a commonly used term in the title or the abstract	p.1	Abstract states: "This study evaluated... over a 7-day period."
		(*b*) Provide in the abstract an informative and balanced summary of what was done and what was found	p.1	Abstract includes Background, Objective, Methods, Results, and Conclusion, summarizing study design, sample (58 athletes), key findings, and recommendations.
**Introduction**				
Background/rationale	2	Explain the scientific background and rationale for the investigation being reported	p.2	Introduction provides comprehensive background on hydration importance, sport-specific challenges in taekwondo, and the research gap.
Objectives	3	State specific objectives, including any prespecified hypotheses	p.2	Introduction provides Study objective in introduction outlines three specific aims.
**Methods**				
Study design	4	Present key elements of study design early in the paper	p.2 (Sec 2.1)	Section 2.1 explicitly states study design: "This study employed a 7-day repeated-measures observational field study design... "
Setting	5	Describe the setting, locations, and relevant dates, including periods of recruitment, exposure, follow-up, and data collection	p.2 (Sec 2.1)	Section 2.1 explicitly states study setting: "conducted at the National Winter Sports Training and Research Base in Beijing, China, June 2023."
Participants	6	(*a*) *Cohort study*—Give the eligibility criteria, and the sources and methods of selection of participants. Describe methods of follow-up<br>*Case-control study*—Give the eligibility criteria, and the sources and methods of case ascertainment and control selection. Give the rationale for the choice of cases	p.2-3 (Sec 2.2)	Section 2.2 and 2.4: whole-team sampling; 66 recruited; 58 athletes completed the full 7-day monitoring protocol and were included in the daily hydration analysis. For training session analyses, 58 athletes completed the technical and tactical training session assessment, 59 completed the physical fitness training session assessment and 49 completed the strength training session assessment.

		and controls<br>*Cross-sectional study*—Give the eligibility criteria, and the sources and methods of selection of participants		
		(*b*) *Cohort study*—For matched studies, give matching criteria and number of exposed and unexposed<br>*Case-control study*—For matched studies, give matching criteria and the number of controls per case	N/A	
Variables	7	Clearly define all outcomes, exposures, predictors, potential confounders, and effect modifiers. Give diagnostic criteria, if applicable	p.4-6	Outcomes clearly defined: hypohydration (USG >1.020 and Uosm >=800), fluid intake, body mass loss, sweat ions... Predictors/confounders: sex, training years, athletic level, education, temperature, intensity.
Data sources/ measurement	8*	For each variable of interest, give sources of data and details of methods of assessment (measurement). Describe comparability of assessment methods if there is more than one group	p.3-6	Each variable detailed: (Sec 2.6) thermo-hygrometer; (Sec 2.7) anthropometrics; (Sec 2.8) 24-hour fluid replacement record recording questionnaire; (Sec 2.9) Clinitek/SMC; (Sec 2.10) Easylyte/Baker equation; (Sec 2.11) digital scale; (Sec 2.12) Firstbeat.
Bias	9	Describe any efforts to address potential sources of bias	p.6 (Sec 2.13)	Section 2.13 addresses measurement bias, information bias, selection bias, and confounding.
Study size	10	Explain how the study size was arrived at	p.2-3 (Sec 2.3)	Section 2.3: a priori power analysis using GPower 3.1.9.7, Wilcoxon signed-rank test, f=0.40, α =0.05, power=0.8, required n=54. Enrolled 66, 58 completed.

Continued on next page

Quantitative variables	11	Explain how quantitative variables were handled in the analyses. If applicable, describe which groupings were chosen and why	p.6 (Sec 2.14)	Section 2.14: non-normal data as median from quantile regression [M(QR)] with Kruskal-Wallis H; normal data as mean ± standard deviation (M±SD) with ANOVA.
Statistical methods	12	(*a*) Describe all statistical methods, including those used to control for confounding	p.6 (Sec 2.14)	Section 2.14 describes: Wilcoxon signed-rank test, Kruskal-Wallis H test followed by Bonferroni pairwise, one-way ANOVA, interpolation regression for missing values.
		(*b*) Describe any methods used to examine subgroups and interactions	p.6 and Results	Subgroup analyses by sex, training schedule, dehydration level, education, training duration, athletic rank using Kruskal-Wallis with Bonferroni pairwise.
		(*c*) Explain how missing data were addressed	p.6 (Sec 2.14)	Section 2.14: "missing values were imputed using interpolation regression."
		(*d*) *Cohort study*—If applicable, explain how loss to follow-up was addressed<br>*Case-control study*—If applicable, explain how matching of cases and controls was addressed<br>*Cross-sectional study*—If applicable, describe analytical methods taking account of sampling strategy	N/A	Not applicable - whole-team sampling was used (all eligible athletes invited), not a probability-based sampling strategy.
		(*e*) Describe any sensitivity analyses	N/A	
**Results**				
Participants	13*	(a) Report numbers of individuals at each stage of study—eg numbers potentially eligible, examined for eligibility, confirmed eligible, included in the study, completing follow-up, and analysed	p.6-8 (Sec 3.1)	Section 3.1: "initially enrolled 66... 58 athletes successfully completed the full 7-day monitoring protocol, with their data included in the final analysis."
		(b) Give reasons for non-participation at each stage	p.2 (Sec 2.2)	Section 2.2: "Eight athletes were excluded due to incomplete data collection (missing ≥1 days of urine samples or fluid intake records)."
		(c) Consider use of a flow diagram	p.3 (Fig 1)	Figure 1 shows study procedure timeline. Participant numbers stated in text (66 enrolled and 58 analyzed) but no dedicated participant flow diagram.
Descriptive data	14*	(a) Give characteristics of study participants (eg demographic, clinical, social) and information on exposures and potential	p.5, 7, 9	Table 2 presents demographics (age, stature, body mass, BMI, Body surface area) by gender, training years, athletic grades and education level. Confounders (temperature, humidity, intensity of training) in Tables 1 and 3.

		confounders		
		(b) Indicate number of participants with missing data for each variable of interest	p.2 (Sec 2.2)	Indicate overall attrition stated, with 8/66 excluded.
		(c) *Cohort study*—Summarise follow-up time (eg, average and total amount)	N/A	
Outcome data	15*	*Cohort study*—Report numbers of outcome events or summary measures over time		
		Case-control study—Report numbers in each exposure category, or summary measures of exposure		
		Cross-sectional study—Report numbers of outcome events or summary measures	P.4-6	Outcome events including fluid replacement, urine indices, sweat ion concentration, body mass loss, energy expenditure
Main results	16	(*a*) Give unadjusted estimates and, if applicable, confounder-adjusted estimates and their precision (eg, 95% confidence interval). Make clear which confounders were adjusted for and why they were included	P.6-11	Results report (a) M(QR) and P-values from Kruskal-Wallis H tests followed by pairwise comparisons with Bonferroni correction for non-normally distributed data,. (b) M±SD and P-values from one-way ANOVA for normal distribution data. Effect sizes and confidence intervals are not consistently reported.
		(*b*) Report category boundaries when continuous variables were categorized	p.8 and 11 (Sec 3.3, 3.5)	Section 3.3 defines hypohydration cutoff: "urine specific gravity > 1.020 and urine osmolality ≥ 800 mOsm/kg." Sec 3.5 defines appropriate rehydration regime cutoff: " urine specific gravity ≤ 1.020", " body mass loss < 2%"
		(*c*) If relevant, consider translating estimates of relative risk into absolute risk for a meaningful time period	N/A	

Continued on next page

Other analyses	17	Report other analyses done—eg analyses of subgroups and interactions, and sensitivity analyses	p.7-11	Subgroup analyses (gender, training years, athletic level, education) in Figures 2-3. Workday vs weekend and training session comparisons reported.
**Discussion**				
Key results	18	Summarise key results with reference to study objectives	p.11 (Sec 4)	First paragraph of Discussion summarizes key findings in relation to study objectives: chronic dehydration, worse on training days, pre-training hypohydration, technical-tactical sessions most severe.
Limitations	19	Discuss limitations of the study, taking into account sources of potential bias or imprecision. Discuss both direction and magnitude of any potential bias	p.12-13	Limitations discussed: (a) the sweat biomarker calculations relied on regional sampling combined with estimation formulas (b) recall bias and social desirability bias of self-reported 24-h recall questionnaire; (c) interference with dietary (d) summer-only data; (e) urine output not measured; (f) no control group.
Interpretation	20	Give a cautious overall interpretation of results considering objectives, limitations, multiplicity of analyses, results from similar studies, and other relevant evidence	p.12	Interpretation is cautious and contextualized: compares to soccer and combat sports, discusses mechanisms, acknowledges observational nature, avoids overstating causality.
Generalisability	21	Discuss the generalisability (external validity) of the study results	p.11-12	Generalisability discussed: specific to Chinese youth taekwondo athletes at national team level; summer-only data; combat sport context may be transferable to similar sports.
**Other information**				
Funding	22	Give the source of funding and the role of the funders for the present study and, if applicable, for the original study on which the present article is based	p.14	Funding section: "The author(s) declared that financial support was received for this work and/or its publication. This research was funded by the Fundamental Research Funds for the Central Universities, The project code is 2024YJSY001."<br>Conflict of interest: "The author(s) declared that this work was conducted in the absence of any commercial or financial relationships that could be construed as a potential conflict of interest. "

*Give information separately for cases and controls in case-control studies and, if applicable, for exposed and unexposed groups in cohort and cross-sectional studies.

Note: An Explanation and Elaboration article discusses each checklist item and gives methodological background and published examples of transparent reporting. The STROBE checklist is best used in conjunction with this article (freely available on the Web sites of PLoS Medicine at http://www.plosmedicine.org/, Annals of Internal Medicine at http://www.annals.org/, and Epidemiology at http://www.epidem.com/). Information on the STROBE Initiative is available at www.strobe-statement.org.
